# Supplementary material for: Factors associated with Onchocerca volvulus transmission after 20 years of community treatment with ivermectin in savanah and forest areas in Central African Republic: A Cross Sectional Study
Source: PLoS Negl Trop Dis. 2024 Dec 16;18(12):e0012325. doi: 10.1371/journal.pntd.0012325 (PMC11684659; doi:10.1371/journal.pntd.0012325)
Supplement: Attachment — Submitted filename: RESPONSE TO REVIEWERS.docx [file pntd.0012325.s001.docx]

**Responses to Reviewers**

We would particularly like to thank all the Reviewers for the importance they attached to our manuscript. Their valuable comments and suggestions have been very helpful in improving the quality of our work. We have tried to respond point by point to these concerns and hope that these answers will satisfy you.

**Reviewer # 1**

1. **Results**

Does the analysis presented match the analysis plan? more analysis to do

We have stepped up our analysis. Take a look at the results in the tables and also in the text

Are the results clearly and completely presented? Incomplete

Results completed

Are the figures (Tables, Images) of sufficient quality for clarity? Tables to improve

Tables improved, please check it

2. **Conclusions**

-Are the conclusions supported by the data presented? yes but incomplete

We've improved our conclusion

-Are the limitations of analysis clearly described? see above

It is done

-Do the authors discuss how these data can be helpful to advance our understanding of the topic under study? Yes but does not not enough describe the oncho bvurden of disease

We've improved our conclusion and onchocerciasis bruden described

-Is public health relevance addressed? Not enough

See the improved text

3. **Editorial and Data Presentation Modifications**

Introduction mortality is not only because of blindness but also because of epilepsy

We agree with this idea and have included it in our reasoning in the manuscript.

O. volvulus should be in italic

Corrections made.

It is mentioned that CAR is among the countries that have made progress in onchocerciasis control and elimination (11,14) Are these ref showing this? I propose to omit this sentence. Most likely since the start of the civil war in 2013 the programme has been very weak.

We removed this sentence as required

How were skin snips read? with an inverted microscope?

Yes. Microscopes acquired as part of the programme. Members of the medical team have been trained in their use by the National Onchocerciasis Control Programme team, with support from the WHO and other partners.

Prevalence of symptoms need to be reported in a Table: for example
Nodules 6.2%

It is reported in Table2. It may go as shown in Table 2?

Blindness 25%: extremely high How was blindness defined?

Yes blindness is particuarly high in Bossangoa district. We considered visual impairment and permanent blindness as blindness

Epilepsy 9.1% also extremely high

Yes, we found it as exactly

Line 263: 99/600 (6.19) have onchocerciasis-associated epilepsy (OAE) status. This should be 99/1600

You are right. Correction made in the text

Ref 10 is a good ref for OAE but not appropriate were it is mentioned now.

Ok, correction made.

More ref about epilepsy in oncho areas need to be included certainly the
ref -Desmet E about prevalence of epilepsy in oncho area in the CAR clos to the DRC border and the ref of Methano about nodding syndrome in that area need to be added

References added. Very interesting references.

Was er a difference in blindness/epilepsy prevalence between forest and Savanna area?

Yes, we extracted data from the 2 districts (savannah and forest). Using a proportion comparison test, we noted that the prevalence of blindness and epilepsy is higher in the savannah zone (Bossangoa) than in the forest zone (Kemo), with p < 0.001.

We head nodding seizures reported in persons with epilepsy?

We didn't note this aspect

4. **Summary and General Comments**

Interesting paper on onchocerciasis in the CAR.

Ok
Very little recent information is currently available about the onchocerciasis situation in the CAR.

Ok

Therefore this paper is very useful.

Ok
The extremely high burden of oncho disease because of blindness and OAE needs to be mentioned in the conclusion.

Ok, conclusion improved with this aspect

This prevalence study was done after an CDTI effort in 2023. Most likely the situation would have been worse before the 2023v distribution.

Certainly

Weakness of the study is that persons with epilepsy were not examined nor very well interviewed about the type of seizures an time of onset of the first seizures.

Respondents were examined by a physician and by 6th year medical students. In areas where onchocerciasis is endemic, epilepsy is well known by the population and has a specific name in local languages.

Are there pigs in the area?

Yes

Do the persons with epilepsy have access to treatment?

Yes

**Reviewer # 2**

1. **Methods**

In the Methods section, there can be some more clarification on what specific skin and eye characteristics researchers looked for (lines 164-165).

The characteristics of the skin and eyes are described in this part of the text.

Additionally, what were the specific disease symptoms in the skin/eyes or skin changes being measured during the community diagnosis process (line 180).

The same symptoms as mentioned above

What was the definition of epilepsy and seizures given to the respondent (lines 183-184)?

In areas where onchocerciasis is endemic, epilepsy and seizures are well known by the population and have a specific name in local languages.

Throughout the Data collection and tools section (Methods), The author needs to clarify the difference an individual and community diagnosis. Both types are currently being described as skin and eye symptoms. Also, if the community diagnosis is currently defined as only skin and eye symptoms, why does the author subsequently include epilepsy and seizures?

The difference is that, for the community diagnosis, when the respondent claims to have signs of skin and eye involvement, a clinical examination is carried out for confirmation.

The community diagnosis includes also epilepsy and seizures.

It is unclear how each of the variables were measured separately to limit confounding factors. If they were not truly seperated (other than just considering each variable on their own), it is unsure how a bivariate analysis would show an appropriate correlation.

In reality, there is no separation of variables. We corrected it in the text

2. **Results**

Does the analysis presented match the analysis plan? Refer to the previously mentioned issues surrounding the chosen statistical analysis methods.

Ok

Are the results clearly and completely presented? To improve organization, "some respondents acknowledged that they had not taken ivermectin during the last distribution campaign" (lines 209-210) should be moved to the Ivermectin uptake and onchocerciasis status section and removed from the Baseline characteristics section. This characteristic should also be moved to the appropriate table.

It is done. See in the text manuscript and Table 3. Table 3 clearly shows that 610 respondents declared that they had not taken ivermectin.

Table 1 includes sex and duration of stay, but these factors are not mentioned in the written results of Baseline characteristics of respondents.

It is now mentioned in the written results

The statement "living in a rural environment appears to protect against..." is misleading based on what is actually being measured (lines 232-233).

If you look at the configuration of the Kémo district, which is in a forest region, the rivers are in urban areas.

Are the figures (Tables, Images) of sufficient quality for clarity? In Table 1, it is unclear what numbers correspond to participants in the hunting and fishing profession. The term "frequency" is incorrect to describe the number of participants, as this word mostly correlates to an instance of disease or event.

In table 1, we replaced the term ‘frequency’ by ‘number’.

In Table 2, what was the study's definition of nodules and blindness (partial or full blindness)? Additionally, did the symptoms of seizures include epilepsy (as epilepsy was previously mentioned as part of the community diagnosis)? What do the numbers in parenthesis represent (is the unit of measure a percent)?

This question is similar to the one we asked about methods, which we answered. We improved the tables for a better interpretation

Need to be consistent throughout all of the tables when including the percentages of participants belonging to each independent variable.

This concerns tables 1 and 2, which we have corrected.

3. **Conclusions**

Are the conclusions supported by the data presented? Given the limitations of the data analysis, it is unclear whether these factors can be considered separate variables contributing to disease transmission.

We have corrected this aspect in the methods section

Are the limitations of analysis clearly described? If proximity to rivers is associated with transmission due to the location of black fly breeding grounds, why did the fishing profession not show any association with transmission?

That's a good observation. We have extracted data on fishermen and indeed there is an association of transmission with this profession as shown in tables 4 and 5 and we have taken account of this in the results and discussion.

Why is male sex not significant in the Kémo district but significant in the Bossangoa district?

Maybe this is due to the activities of men in forest areas and savannah areas. In the forest zone it's much more hunting and gathering. Maybe this is due to the activities of men in forest areas and savannah areas. In the forest zone it's much more hunting and gathering.

4. **Editorial and Data Presentation Modifications**

The tables should be modified to include consistent units of measurement, i.e. percentages of participants belonging to each variable.

It is done

Kémo and Bossangoa also were referred to as the forested area and the savannah area, respectively, throughout the study. For consistency, the author should refer to each of these areas by one name (either the health district name or the geographical name) in the methods, results, and conclusion section.

We wanted to highlight whether the situation differs in savannahs and forests

5. **Summary and General Comments**

There are some weaknesses surrounding the current data analysis. This issue can be improved by an in-depth discussion regarding study's conclusions and limitations or by finding a different way to separate the independent variables.

We have improved our conclusions, which you will appreciate
