# Supplementary material for: Factors associated with Onchocerca volvulus transmission after 20 years of community treatment with ivermectin in savanah and forest areas in Central African Republic: A Cross Sectional Study
Source: PLoS Negl Trop Dis. 2024 Dec 16;18(12):e0012325. doi: 10.1371/journal.pntd.0012325 (PMC11684659; doi:10.1371/journal.pntd.0012325)
Supplement: Attachment — Submitted filename: RESPONSE TO REVIEWERS.DOCX [file pntd.0012325.s002.DOCX]

**Responses to Reviewers**

We would particularly like to thank all the Reviewers for the importance they attached to our manuscript. Their valuable comments and suggestions have been very helpful in improving the quality of our work. We have tried to respond point by point to these concerns and hope that these answers will satisfy you.

**Reviewer # 1**

Line 72: “the study showed 73 that in both the savannah and the forest, men are particularly at risk, not only because of their 74 activities near rivers, but also because they do not protect their bodies as women do” But men also were less likely to take ivermectin! This seems to be much more important.

This is what we found out on site.

Line 95 simuliums should be Simuli (or blackflies) who reproduce : ok, correction made

Line 99 the microfilariae do not reproduce. ok, correction made

Line 100 A genuine public health problem in terms of morbidity, onchocerciasis is observed in three WHO regions. Sentence is not ok I propose to state “Onchocerciasis is observed in three WHO regions:

ok, correction made

Line 131 Are there people rearing pigs? Yes !

Line 110 Some of the consequences of this disease on the lives of people in endemic areas 110 remain a cause for concern (3,4,7–10).

Line 117 "treatment increased from 119 million in 2015 to 132 million in 2016” … million persons treated? Yes !

Line 119 “The Central African Republic (CAR) has endemic onchocerciasis in 20 health districts in 120 savannah and forest areas.” Should be “In the Central African Republic (CAR) onchocerciasis is endemic in 20 health districts in 120 savannah and forest areas. ok, correction made

Line 161 remove For this study, ok, correction made

Line 172 Prevalence of onchocerciasis is determined by individual and community diagnosis. Should be was. ok, correction made

Also a skin sample is taken should be was taken. ok, correction made

It is not clear when skin snips were taken. On everybody or only when skin or eye manifestations suggested onchocerciasis. Only when skin or eye manifestations suggested onchocerciasis.

Need to explain this in the methods. Ok

Line 180 community diagnosis is epidemiological??? Not clear. Through a clinical examination.

Line 181 unclear rewrite. Describe how you assessed symptoms and signs. Symptoms and signs were assessed by 6th year medical students and members of district health management teams trained in the management of onchocerciasis in 2023.

Line 191: how was onchocerciasis infection status determined ? based on presence or absence of mf in skin snips? based on presence of mf in skin snips

Line 206 omit to this study. Ok, correction made

Line 208 work should be worked. Ok, correction made

Line 209 omit Table 1 shows the other characteristics of the participants. Refer to (Table1) after The average age of participants was 31.2 years (± 6.5). Ok, correction made

Table 1 improve lay out. You should rounding the number to one decimal place. Ok, correction made

Line 213: 357 had onchocerciasis How was this defined symptoms of onchocerciasis?

Table 2 Improve lay out. Title onchocerciasis according to signs and symptoms but Onchocerciasis status based on skin snip results? No, based on signs and symptoms

Need a separate Table for prevalence of symptoms

Table 4 and 5 transmission of onchocerciasis should be transmission of Onchocerca volvulus. Ok, correction made

Line 233 omit after several years of community distribution of ivermectin. Ok, correction made

Line 237 “and the fishing profession” should be “and fishing”. Ok, correction made

Line 237-9 “In forested areas, profession other than fishing plays no role in onchocerciasis transmission, and living in a rural environment or hunting/agriculture appears to protect against onchocerciasis transmission..” Not clear I propose to state only fishing was associated with onchocerciasis. Ok, correction made

Line 261 omit studied. Ok, correction made

Line 262 and 38.12% (610/1600), which means that strategies need to be developed to reach people in remote 263 areas. Unclear, rewrite. Ok, correction made

Line 271 Similar proportions were.. should be A high prevalence of epilepsy was also. Ok, correction made

Line 279 -80 explanation is not clear. Ok, correction made

Line 284-5 rewrite sentence. Ok, correction made

Line 282-83 men were less likely to take ivermectin. Please mention this. This seems to be a more important factor than the way of covering the body! Ok, correction made

Line 297 high prevalence of blindness : no prevalence in the results. Ok, correction made

298 blin should be blind. Ok, correction made

Replace very high by high. Ok, correction made To calculate the population prevalence you should include in the denominator also the children < 5 years. Do you have this number? No
